# Our Experience With Transduodenal Surgical Ampullectomy

**DOI:** 10.7759/cureus.21467

**Published:** 2022-01-21

**Authors:** Samer Dbouk, Nagham Bazzi, Lea Daou, Zaynab Shaalan, Ali Choukr

**Affiliations:** 1 General Surgery, Al Zahraa Hospital University Medical Center, Beirut, LBN; 2 Faculty of Medical Sciences, Lebanese University, Beirut, LBN; 3 Faculty of Health Sciences, Beirut Arab University, Beirut, LBN

**Keywords:** ampulla, transduodenal ampullectomy, whipple procedure, endoscopic papillectomy, tumors of the ampulla

## Abstract

Tumors of the ampulla of Vater are uncommon lesions accounting for only 0.5% of the gastrointestinal tumors. A total of three techniques for ampullary tumors (AT) exist: endoscopic papillectomy (EP), surgical transduodenal ampullectomy (TDA), and partial pancreatoduodenectomy (PD). Scarce articles report the outcomes of TDA.

Two Arabic men presented to the hospital with epigastric pain and were subsequently diagnosed with AT. The TDA was performed a few days after the diagnosis. The first patient underwent a partial gastrectomy on the eleventh postoperative day. The second patient underwent endoscopic pyloric re-opening on the eleventh postoperative day to relieve gastric obstruction.

Both patients started tolerating food and were discharged home. Further randomized controlled studies assessing long-term complications, efficacy, and efficiency of TDA are now warranted.

## Introduction

Tumors of the ampulla of Vater are relatively uncommon lesions accounting for only 0.5% of gastrointestinal tumors [1]. Owing to the high propensity for malignant transformation, excision of the ampullary tumor (AT) is crucial [2].

The AT could be resected with endoscopy or surgery. The endoscopic techniques are endoscopic papillectomy (EP), or "the larger" endoscopic ampullectomy (EA). There are three surgical techniques ranging from the simplest to the most complex: transduodenal ampullectomy (TDA), surgical ampullectomy with complete resection of the common bile duct, and partial pancreatoduodenectomy (PD) (Whipple's procedure) [3]. The preoperative choice of the technique is not yet fully elucidated [1].

Benign lesions with a low rate of malignancy are preferably treated with endoscopic procedures [1]. However, malignant lesions with clear invasion to the duodenum or the pancreas seen on preoperative endoscopic ultrasound (EUS) or those with a suspicious lymph node seen on axial imaging or positron emission tomography (PET) scan are usually managed with pancreatoduodenectomy and level II dissection of the mesopancreas [3].

The transduodenal ampullectomy is performed in benign lesions not amenable to endoscopic resection post sphincterotomy, and large tumors or lesions with endobiliary extension not accessible with endoscopy. Compared to PD, TDA has lower morbidity and mortality but a higher recurrence rate due to limited tissue resection and lymph node dissection [4]. Therefore, a ratio assessing the risks and benefits for each technique along with patients' characteristics is estimated.

Despite the increased interest in performing TDA, the clinical nuances and outcomes of this surgery have not been described clearly, and till now, there's no standardized approach for the technique choice. In this article, we report the presentation, complication, and management via TDA in two patients.

## Case presentation

Case one

**Figure 1:**
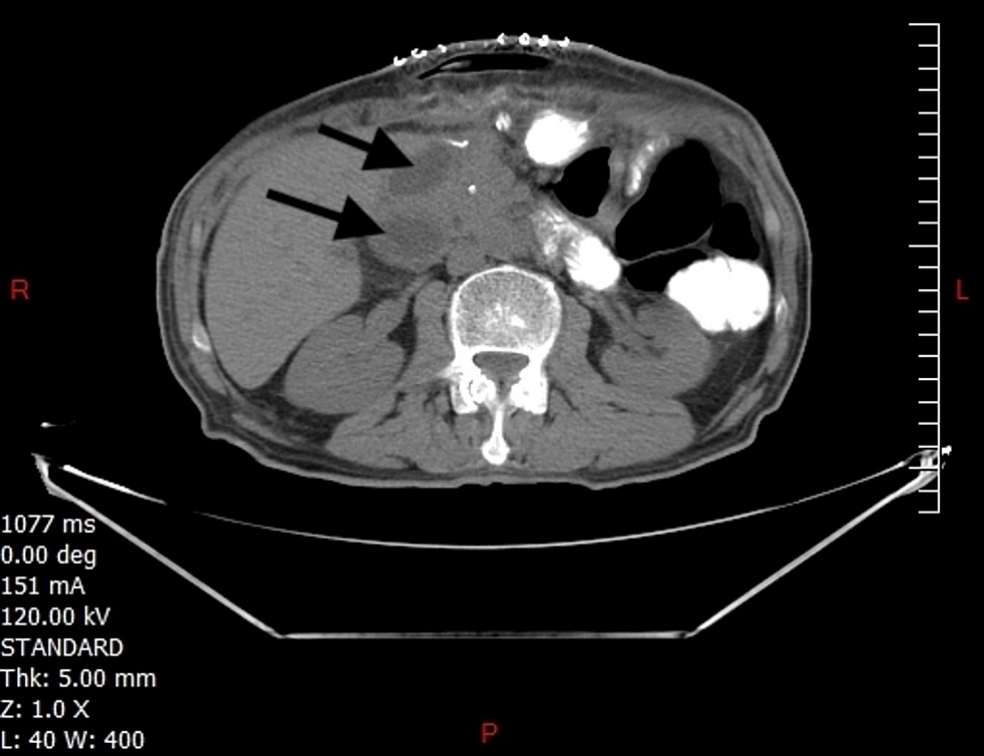
Abdominal CT scan with oral contrast showing biliary ducts dilation (arrows)

A sixty-seven-year-old Arabic man, who is a non-smoker, hypertensive, diabetic, and hyperlipidemic, presented to the hospital with severe epigastric abdominal pain. Physical examination and laboratory tests were unremarkable except for a mild elevation of the pancreatic enzymes. Liver enzymes and CA19-9 levels were normal. An abdominal CT scan revealed intra- and extra-hepatic biliary tree dilation (Figure 1).

The magnetic resonance cholangiopancreatography (MRCP) showed dilated biliary/pancreatic ducts without stones. The endoscopic retrograde cholangiopancreatography (ERCP) combined with a EUS showed mild double duct dilation with a large edematous friable fungating lesion at the level of the ampulla with no duodenal wall invasion, no pancreatic extension, and no lymph nodes detected. Biopsies taken have confirmed the presence of a benign ampulloma. Subsequently, a sphincterotomy with endobiliary stent insertion was performed.

**Figure 2:**
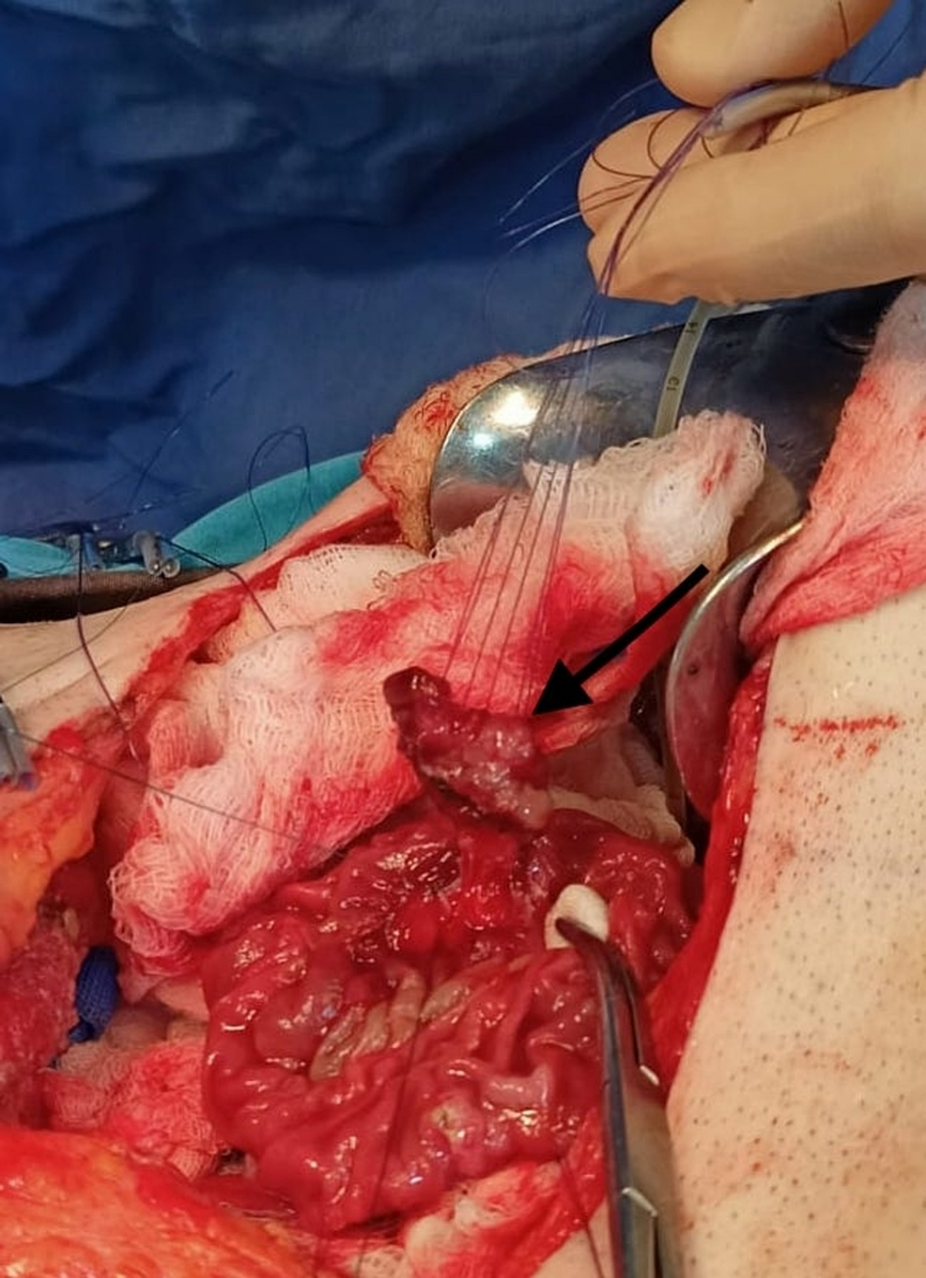
Intra-operative image of surgical transduodunal ampullectomy showing the ampulloma (arrow)

A TDA with intraoperative frozen sections was performed. A complete resection of the ampulla of Vater, the sphincter, the distal part of the common bile duct (CBD), Wirsung duct as well as the duodenal wall around the papilla was done and sent to the histopathology section (Figure 2).

**Figure 3:**
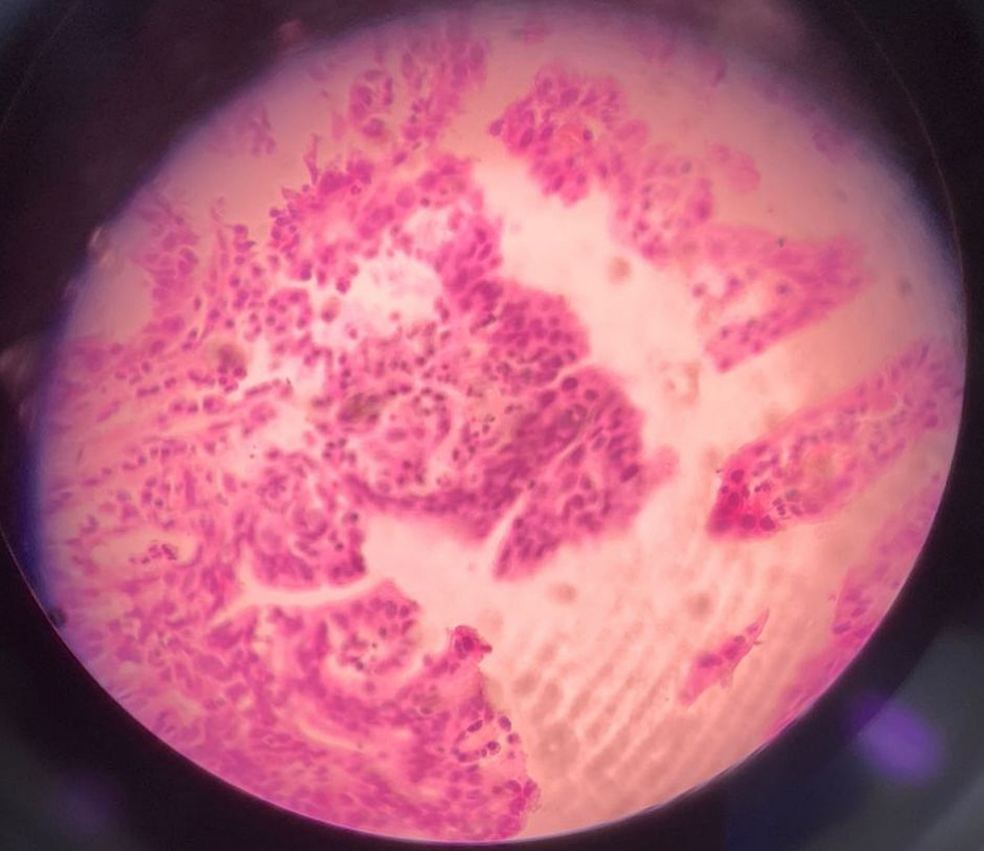
Ampullary adenoma with low-grade dysplasia

On frozen section, the ampulla was grossly expanded and widened with polypoid components; no hard or infiltrative areas were found. Surgical margins were unremarkable except that the CBD margin appeared edematous and thickened. The main pathology was the presence of adenomatous glands along with wide areas of low-grade dysplasia in lining epithelium and glands (Figure 3).

The lesion was limited to the mucosa with a mildly inflamed submucosa. The CBD was re-trimmed until reaching negative margins, and both the CBD and pancreatic duct were re-implanted in the duodenum.

On the fifth day, clear fluid diet was started. Subsequently, the patient reported having persistent vomiting. The upper gastrointestinal series showed huge gastric dilation with slow emptying of contrast at the level of the bulb of the duodenum (D1) and a mild reflux into the common bile duct, as well as gas with gastroparesis and failure of improvement on intravenous prokinetics. As a result, a partial gastrectomy with a Billroth II anastomosis was performed on the 11th post-operative day. The patient was started afterwards on full fluid diet, tolerated well and was discharged 17 days after the initial ampullectomy.

Case two

A seventy-eight-year-old Arabic man, who's a non-smoker, hypertensive, and diabetic, presented to the hospital with weight loss, black tea-colored urine, jaundice, and vomiting for the past three months.

**Figure 4:**
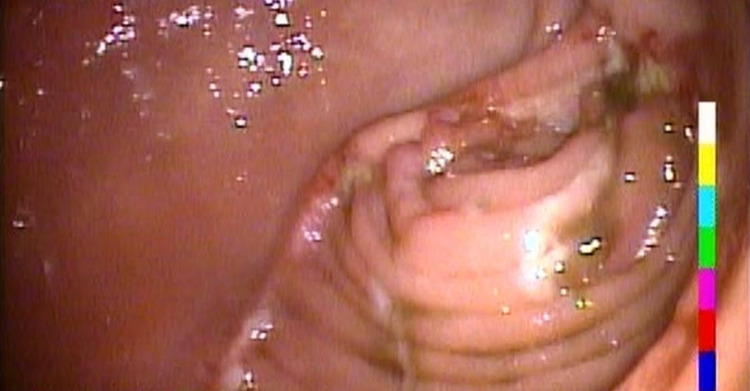
Ampullary tumor visualized on ERCP ERCP - endoscopic retrograde cholangiopancreatography

The CT scan showed biliary dilatations with pancreatic duct dilatation but no mass was found in the pancreatic head. The common hepatic artery was completely thrombosed. The liver received the arterial blood supply from collaterals arising from the gastroduodenal and superior mesenteric arteries. The patient's abdominal vessels were all calcified. The MRCP revealed a dilated CBD (eight millimeters) with no filling defect. On ERCP combined with EUS a peri-ampullary lesion was found with no direct invasion to the duodenal wall, no pancreatic invasion and no endobiliary extension (Figure 4).

Biopsies were taken and showed an adenoma with high grade dysplasia.

**Figure 5:**
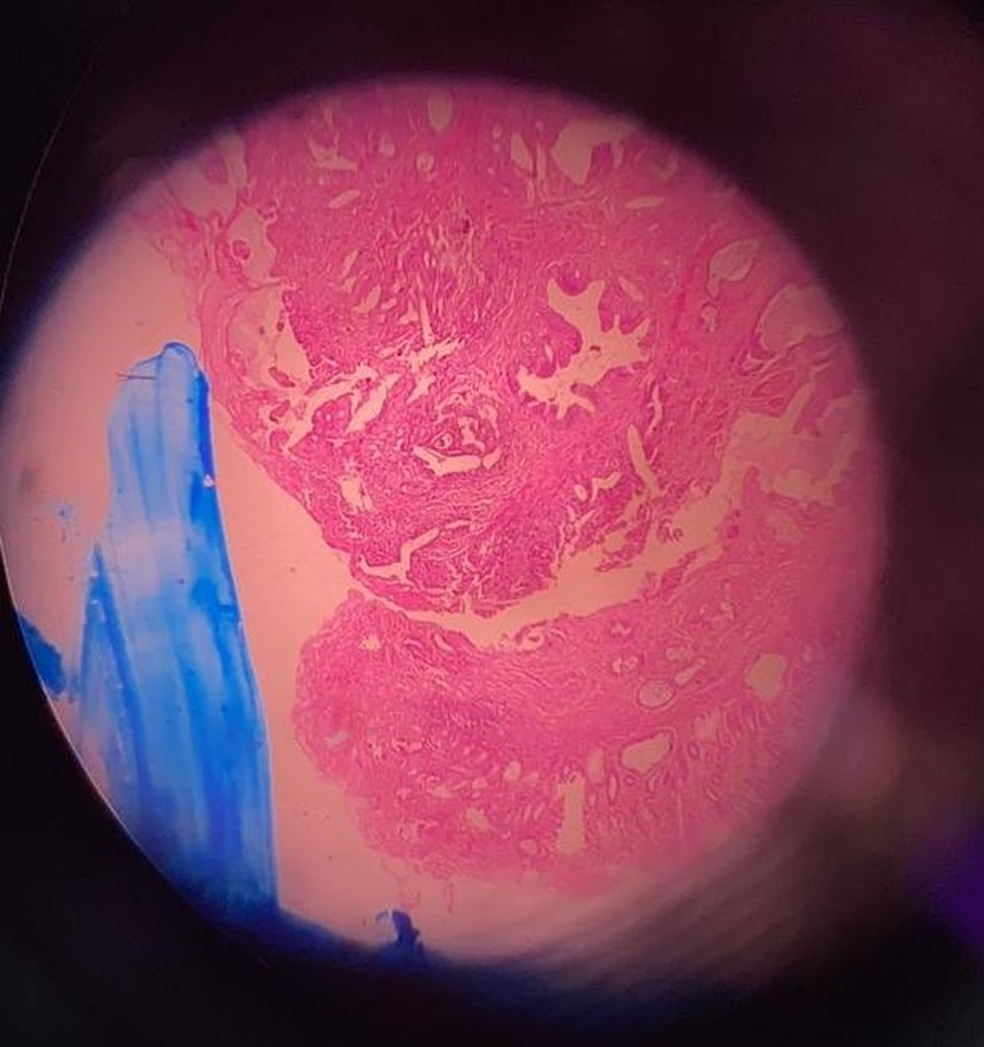
Ampullary adenocarcinoma of intermediate grade

A transduodenal ampullectomy with total removal of the ampullary complex, reconstruction of the biliary and pancreatic duct over the duodenal wall, and stent placement were done. The frozen section confirmed the presence of an adenocarcinoma mostly of intermediate grade and focal high-grade component (Figure 5).

The lesion presented as a focal firm mass at the ampulla and measured 1×1×0.5 cm. The tumor infiltrated the mucosa, submucosa, and the superficial muscularis propria but without vascular invasion. The CBD and pancreatic duct margin sample were exempt of malignant cells and the ductal anatomy was re-implanted. A pyloric exclusion was performed with vertical mattress suturing of the distal antrum with thick monofilament sutures and a side to side Billroth II type gastrojejunostomy was performed.

Due to the complete calcification of the supraceliac aorta and splenic artery, the liver could not be revascularized after a pancreatoduodenectomy (PD) Whipple's procedure.

**Figure 6:**
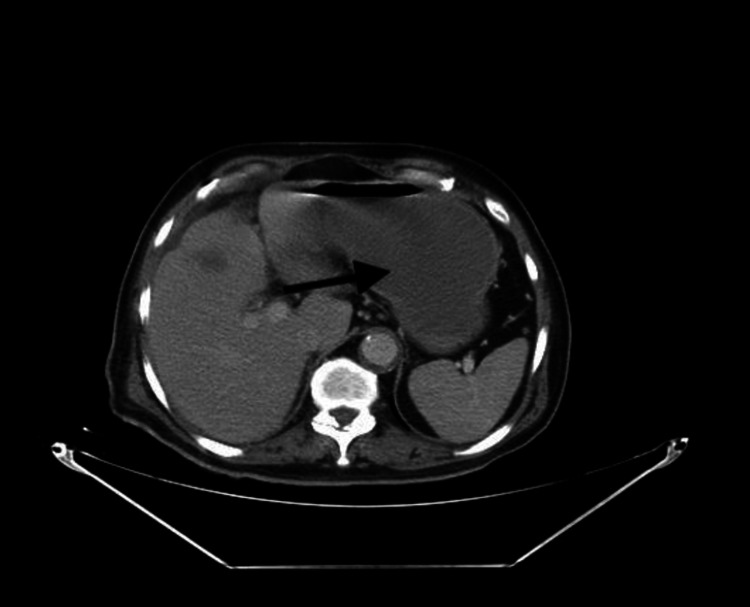
Abdominal CT scan showing distended stomach (arrow) 10 days post-operatively

On the 10th post-operative day, the patient was not tolerating food. The upper gastrointestinal series and the abdominopelvic CT scan showed gastroparesis and distended stomach, respectively (Figure 6).

Thus, a gastroscopy was performed on the 11th post-operative day and confirmed a stenotic pylorus. Consequently, endoscopic pyloric re-opening was performed. The patient was discharged on the 18th post-operative day.

## Discussion

This article summarizes the findings of two rare cases of ATs; a benign ampulloma and a periampullary malignant lesion.

Classically, TDA is geared towards low grade dysplastic ampullary adenomas, whereas EP is performed in periampullary neoplasms sparing the common bile duct and the pancreatic duct [5, 6]. According to the European Society of Gastrointestinal Endoscopy (ESGE) guidelines for ampullary tumors, EP should be adopted in high grade dysplasia with a size between 20 and 30 mm and bile or pancreatic duct progression measuring ≤20 mm. Whereas TDA should be considered in Tis cancer, adenoma demonstrating bile or pancreatic duct progression measuring >20 mm, and adenoma where EP would present technical difficulties due to diverticulum or a large size measuring ≥40 mm [7].

The delayed gastric emptying reported in case two could be induced by worsened diabetes post-surgery. An inverse relation between glucose concentration and gastric emptying exists, and acute variation in glucose concentration significantly affects gastric emptying [7]. The patient in case two was initially diabetic, and his diabetes was very difficult to control during the post-operative period. His blood glucose readings in the first few post-operative days reached values such as 310, 328, and 387 mg/dl at times. Surgical patients are known to develop hyperglycemia post-operatively due to hypermetabolic stress response causing increased glucose production and insulin resistance, which is probably what happened in this patient.

The partial gastrectomy performed in the first patient has hampered the endoscopic follow-up of the patient’s course. Therefore, axial imaging and Ca19-9 levels were used to follow the disease progression.

Kim et al. reported the recurrence of an AT post-TDA [8]. The complications post TDA were delayed gastric emptying (0.04%), wound dehiscence (0.04%), passage disturbance (0.04%), and pancreatitis (33.33%) [8]. Older age, type 2 diabetes mellitus, depression, and smoking are highly linked with complicated TDA [8,9].

Scarce articles describe the TDA procedure; however, EP is widely expanded in the literature [8]. The EP is preferably chosen due to the absence of skin scar post-intervention and the quick return to normal life [8].

## Conclusions

Surgical transduodenal ampullectomy is rarely performed in our country. The gastroparesis post-ampullectomy reported in the two cases suggests combining gastric drainage to the surgery. None of the patients developed biliary or pancreatic complications. Further randomized control studies assessing long-term complications, efficacy, and efficiency of TDA are now warranted.

## References

[1] Jung YK, Paik SS, Choi D, Lee KG (2021). Transduodenal ampullectomy for ampullary tumor. Asian J Surg.

[2] Linn YL, Wang Z, Goh BK (2021). Robotic transduodenal ampullectomy: case report and review of the literature. Ann Hepatobiliary Pancreat Surg.

[3] Schneider M, Büchler MW (2021). [Ampullary neoplasms: surgical management]. Chirurg.

[4] Hong SS, Han SS, Kwon W (2021). Comparison of oncologic outcomes between transduodenal ampullectomy and pancreatoduodenectomy in ampulla of Vater cancer: Korean multicenter study. Cancers (Basel).

[5] Nappo G, Gentile D, Galvanin J (2020). Trans-duodenal ampullectomy for ampullary neoplasms: early and long-term outcomes in 36 consecutive patients. Surg Endosc.

[6] De Palma GD (2014). Endoscopic papillectomy: indications, techniques, and results. World J Gastroenterol.

[7] Sekine M, Watanabe F, Ishii T (2021). Investigation of the indications for endoscopic papillectomy and transduodenal ampullectomy for ampullary tumors. J Clin Med.

[8] Kim J, Choi SH, Choi DW, Heo JS, Jang KT (2011). Role of transduodenal ampullectomy for tumors of the ampulla of Vater. J Korean Surg Soc.

[9] Paluri R, Kasi A Ampullary cancer. https://www.ncbi.nlm.nih.gov/books/NBK555958/.

